# Assessment of Park Paths and Trails for Physical Activity Promotion among Older Adults in Saudi Arabia: Feasibility and Future Directions

**DOI:** 10.3390/healthcare12161572

**Published:** 2024-08-08

**Authors:** Tracy Chippendale, Hadeel R. Bakhsh, Najat A. Alhaizan, Bodor H. Bin Sheeha

**Affiliations:** 1 Department of Occupational Therapy, Steinhardt School of Culture, Education, and Human Development, New York University, 82 Washington Square East, 6th Floor, New York, NY 10003, USA; tlc223@nyu.edu; 2 Department of Rehabilitation Sciences, College of Health and Rehabilitation Sciences, Princess Nourah Bint Abdulrahman University, P.O. Box 84428, Riyadh 11671, Saudi Arabia; naalhaizan@pnu.edu.sa (N.A.A.); bhbinsheeha@pnu.edu.sa (B.H.B.S.)

**Keywords:** physical activity, health promotion, chronic disease, occupational therapy, public parks

## Abstract

Chronic diseases affect more than 80% of older adults. One modifiable risk factor for secondary prevention is physical activity. Public parks are an essential resource for physical activity, but environmental features may limit participation. Our aims were (1) to assess the feasibility and utility of the Path Environment Audit Tool (PEAT) for use in a larger-scale study focused on older adults and (2) to examine the physical activity-promoting features of five public parks. Methods: A descriptive study design was used to assess five public parks in Riyadh, Saudi Arabia, from April to June 2022. Additionally, process, resource, and management assessments were also conducted. Results: The PEAT was easy to use, but additions are recommended to increase relevance to older adults with chronic disease. Descriptive analyses revealed positive features, such as adequate lighting, but other features such as path slope were more variable. Conclusions: Study findings can guide the future use of the PEAT for older adult participants and inform environmental modifications.

## 1. Introduction

Chronic disease is a major cause of death and disability worldwide. In the United States, more than eight in ten older adults have a chronic disease [1]. Globally, Europe has the highest burden of chronic diseases, which are responsible for 86% of all deaths [2]. In Saudi Arabia, a sharp increase in chronic and lifestyle-related diseases such as coronary heart disease, cancer, obesity, diabetes, and hypertension have been recorded [3]. At present, cardiovascular disease is the leading cause of death around the world [4]. Risk factors for cardiovascular disease and type 2 diabetes, such as high body mass index (BMI), elevated fasting glucose levels, and low physical activity levels, are prevalent in many countries around the world, and in some countries, they are on the rise [3,4,5,6]. Moreover, globally, there are a growing number of older adults, a population at a higher risk of developing chronic diseases [7]. 

To address these public health concerns, the Healthy People 2030 initiative in the United States includes objectives related to the number of adults and older adults who meet the minimum guidelines for physical activity and a reduction in new cases of diagnosed diabetes in the population [8]. In the United Kingdom, one of the goals of the National Health Service’s long-term plan is to provide education and exercise programmes to more patients with heart disease to help prevent premature death [9]. Saudi Arabia’s Vision 2030 includes a strategic quality of life and health program [10]. One program objective is health promotion and prevention initiatives to mitigate health risks associated with preventable diseases [7,10,11]. Rehabilitation therapists are well suited to move this work forward and promote lifestyle changes including increased physical activity participation due to their expertise in client-centred practice, environmental modification, and the promotion of healthy habits and routines [12,13,14,15,16]. Examples of chronic disease self-management programs include multicomponent programs that address the needs of people with multiple chronic conditions [17,18], as well as programs that target the needs of people with a specific chronic condition, such as diabetes [19,20,21]. An important component of chronic disease self-management programs is goal setting to help participants increase physical activity participation and improve their health-related quality of life [18,22].

The Ecology of Human Performance Model [23] is an interdisciplinary framework that is highly relevant to health promotion and chronic disease self-management programming. The model highlights the importance of the environment in task performance and describes the relationship among the person, context, task, and performance. Further, the model highlights intervention approaches including alter, adapt/modify, and prevent, which can be used to promote participation in meaningful activities. Alter interventions include finding the best match between a person’s ability and their context (e.g., matching a person’s physical abilities to a specific park), whereas adapt/modify interventions include making changes to the context or task (e.g., advocating for modifications to an existing park). Both alter and adapt approaches can be used to facilitate increased participation in leisure time physical activity. 

Consistent with the tenets of the Ecology of Human Performance Model [23], there is a growing body of literature on the impact of the environment on physical activity, including evidence from studies supporting the benefits of neighbourhood walkability and park availability [24,25,26,27,28]. The relationship between specific components of the social and physical environment and physical activity participation among people at risk for or with chronic diseases has also been explored [29,30,31]. A lack of family, friends, and healthcare provider support, a lack of motivation, not enjoying physical activity, and a lack of facilities have been identified as barriers to physical activity participation [6,32,33,34]. Among older adults, barriers to outdoor physical activity have been identified and include extreme temperatures as well as features of the built environment, such as inclined surfaces, uneven terrain, a lack of available walking paths, the quality of sidewalks, safety from traffic, lighting, and outdoor stairs [34,35,36]. Adapting physical environments and methods of participation have been shown to be key in maintaining active participation in leisure activities for older adults [36]. Consequently, prior research highlights the importance of the social environment and the availability of accessible facilities and their motivating features regarding the promotion of physical activity.

Prior studies reveal that parks and walking trails are an essential resource for leisure-time physical activity [37,38,39,40]. Further, parks may also serve as a resource to promote social participation and social cohesion [41]. However, not all parks are equal, as characteristics and features of parks can influence their use and therapeutic benefits. For example, park features and amenities (e.g., lighting, bathroom availability, exercise equipment), the condition of those features (e.g., condition of path surfaces, bench and bathroom cleanliness), access to and within the park, and the park’s aesthetics (e.g., litter, odour) can all impact its use [42,43].

Recently, researchers have explored older adult’s preferences regarding public parks and walking trails [44,45]. Their findings include a preference of older adults for features such as trees and nature, flat walking surfaces, seating, shade, adequate car parking, and information signs. Given that common activities performed by older adults in parks include walking and spending time with grandchildren, multigenerational features including play equipment were shown to be desirable. Other elements of parks deemed to be important include the conditions of walking paths and peaceful well-maintained spaces away from traffic and close to home [45]. Regarding the promotion of physical activity specifically, nature, water views, and shady trees were ranked most important by older adults [44]. 

There are several existing audit tools designed to examine features of parks that promote physical activity participation including the Community Park Audit Tool (CPAT), the Physical Activity Resource Assessment (PARA) instrument, and the Quality of Public Open Space Tool (POST) [46]. However, the Path Environment Audit Tool (PEAT) is unique in its focus on walking paths/trails [43], which are a relevant resource for physical activity for older adults. Other available measures that assess park environments for the promotion of physical activity focus primarily on playgrounds and sport facilities, which are geared towards children and adolescents [46]. 

The current context in Saudi Arabia highlights the timeliness and relevance of examining accessibility and physical activity-promoting features of public parks. Saudi Arabia is the largest country in the Middle East, covering an area of approximately 2.15 million square kilometres [47]. As of 2023, the population of Saudi Arabia is estimated to be around 32 million people [47]. Although Saudi Arabia currently has a low percentage (2.8%) of adults age 65 and older, the transition to a society with a high concentration of older adults is anticipated by 2042 [48]. Further, non-communicable diseases including cardiovascular disease and diabetes are the main causes of years lived in poor health and premature death in the country. To support the growing older adult population and to facilitate healthy longevity, physical environments and infrastructure have been identified as a priority [48]. 

Moreover, the capital city of Riyadh is the largest city in Saudi Arabia and has undergone rapid expansion; with its mix of modern infrastructure and cultural heritage, it serves as an ideal location to evaluate the effectiveness of public spaces in promoting physical activity. There is little infrastructure for pedestrians, affecting the city’s walkability. However, “greening” of the city by increasing the number of trees and public parks is an initiative currently underway, such as King Salman Park and The Sports Boulevard, with the purpose of enhancing the health and quality of life of local residents [49]. Noteworthily, Riyadh has a very dry desert climate, compared to other regions in the country, and can experience temperature extremes, highlighting the need for outdoor spaces that can accommodate weather-related influences [49]. Although this study targets parks in Saudi Arabia specifically, findings related to implications for future use for community-dwelling older adults who have chronic diseases can apply to other geographic locations.

Research highlights the importance of the environment regarding the promotion of physical activity [24,25,26,27,28], and park features that encourage physical activity have been established [42,43]; little is known about paths/trails in existing public parks [50,51] and whether they meet the physical activity needs of older adults with chronic diseases. Although the accessibility of public spaces has been examined in Saudi Arabia, the focus has been on indoor spaces [52,53]. To our knowledge this is the first study to examine outdoor spaces. Prior to conducting a larger-scale study of parks in Riyadh regarding their physical activity-promoting features, a feasibility study is warranted. Feasibility studies set the stage for larger-scale studies to ascertain whether something is doable, if a project or study should proceed, and if so how [54,55]. Feasibility studies include a process, resource, and management assessment, and include descriptive analyses with the goal of informing a larger-scale study. Although the Path Environment Audit Tool (PEAT) has been used in prior studies and has undergone reliability and validity testing [46,56,57], prior research has not focused on the relevance of the tool to older adults nor has the PEAT been used in the context of a region with a hot climate. Further, scalability will incorporate Occupational Therapy (OT) students for data collection, highlighting the need to establish the ease of use and the best method of recording data. Therefore, the purpose of this study was (1) to assess the feasibility and utility of the Path Environment Audit Tool (PEAT) [43] regarding its potential for future use in a larger-scale occupational therapist-led study, focused on the physical activity needs of older adults, and (2) to conduct a preliminary assessment of the physical activity-promoting features of paths/trails in five public parks in Riyadh using descriptive analyses. 

## 2. Materials and Methods

### 2.1. Design

A descriptive study design was used to examine the features of five public parks regarding their physical activity-promoting features in Riyadh city from April–June 2022. A process, resource, and management assessment [55] was used to assess the feasibility and utility of the PEAT measure [43,51] for its potential use in a larger-scale occupational therapist-led study focused on the physical activity needs of older adults. In this study, we define feasibility and utility of the tool as the ease of use and its potential for applicability to the older adult population, including those living in a hot weather climate such as that of the Middle East.

### 2.2. Measures

The Path Environment Audit Tool (PEAT) [51] is an audit instrument developed to objectively assess the characteristics of trails and paths that may influence physical activity. PEAT operates on the principle that the environmental characteristics of trails can significantly influence their use for physical activities such as walking, biking, and jogging. By systematically evaluating these characteristics, PEAT helps identify features that promote or hinder trail use, thereby informing improvements and interventions [51]. PEAT evaluates trails and paths based on three main categories: design features (11 items, some with sub-items), amenities (16 items, some with sub-items), and maintenance/aesthetics (7 items). Each category comprises several items that are evaluated through a combination of binary (yes = 1/no = 0) and 4-point ordinal scales (none = 0, a little = 1, some = 2, a lot = 3) [51]. A 6-item category for roads that intersect trails is a feature that typically occurs at linear parks and is evaluated through a 5-point scale from “very poor = 0” to “excellent = 4”. 

The PEAT has been shown to be a valid and reliable tool, with moderate-to-high inter-rater reliability for its primary items (k-values: 0.46–0.71; observed agreement: ≥81%), and with a strong agreement between PEAT audits and GPS-derived measures (0.77; k-values: ≥0.57) [46,51,56,57]. However, noteworthily, the tool was not specifically designed for older adults or those with chronic diseases, and to our knowledge, it has not yet been used in the context of a hot weather climate [46]. The user manual and PEAT tool can be accessed from Perelman School of Medicine, University of Pennsylvania’s website (https://www.med.upenn.edu/beat/peat-materials.html, accessed on 15 November 2021).

### 2.3. Procedures

The research team consisted of occupational therapists and one physical therapist, who are academically trained in accessibility. However, to ensure accuracy regarding the use of the PEAT measure, the research team reviewed the PEAT manual and scoring sheet in detail and sought clarification from the developer of the measure through follow-up questions. The five parks selected for the study were purposively sampled based on their popularity (as per online reviews) and location in the middle of neighbourhoods/districts that represented different geographical locations in the city of Riyadh (north, east, south, and west). A hand-held GPS App, Strava, installed in iPhone iOS, was used to map out the paths. A tablet computer with an Excel spreadsheet was used to record responses to each item in the audit tool.

Select members of the research team worked in pairs to complete the PEAT [43] audits of each path segment in the five parks. Prior to the start of data collection, the team members involved in conducting the audits completed a practice trial in one park and brought questions they had back to the rest of the research team. Consistent with the PEAT manual [43], the start of a new segment was determined (1) when there was a change in surface type, (2) when the path had an intersecting trail or road, (3) if the trail width changed by more than 10%, or (4) for long paths without any surface changes or intersecting paths/roads, a distance of approximately 500 m was used to divide the path into segments. Short segments were aggregated to approximate a 500 m distance. 

### 2.4. Analysis

Descriptive statistics including means, standard deviations, and frequencies were used to examine park features. For the presence (yes/no) of specific park features, the proportion of segments in each park with the feature was examined, and a percentage across all parks was also calculated. Quantitative measures pertaining to design features, condition of amenities, and level of maintenance were reported as means and standard deviations across all segments in each park. IBM SPSS Statistics for Windows, version 24 (IBM corp., Armonk, NY, USA), was used for the analyses [58].

## 3. Results

### 3.1. Utility for the Older Adult Population, Process, Resource, and Management Assessment

Two members of the research team conducted the audits of each path segment collaboratively. The PEAT was straightforward and easy to use. Only a few questions arose from the practice audit that were then brought back to the rest of the study team. The amount of time taken to complete the audits ranged from fifteen minutes to an hour and forty minutes for each park, depending on the park size and the number of features present. This timeframe did not include travel time to the parks. In addition, specific to the location of data collection, although most features of the audit were appropriate in a Middle Eastern context, some were not. Examples include questions pertaining to the presence of dogs and emergency call buttons (see the Discussion Section for additional details). 

### 3.2. Descriptive Analyses of Physical Activity-Promoting Features of Five Parks

Parks had anywhere from one to six segments, with the distance of each segment ranging from 400 to 660 m. The results of the descriptive analysis to compare the presence of park features can be found in Table 1. Table 2 includes the descriptive results for quantitative measures pertaining to design, amenities, and trail maintenance. The description of the location and size of park is provided in Table 3, and a detailed description of park features is provided in Supplementary Material S1. As per the results, the five parks contained numerous features that promote participation in physical activity. Key strengths included clear, unobstructed walking paths, sufficient site distance, and adequate lighting found in all five parks. Although walking paths ran parallel to roads, there were buffer areas distancing people from traffic in each park, providing a sense of safety and distance from cars. All but one of the parks provide opportunities for scenic views. Each of the parks had parking lots. However, the number of parking spots varied across the parks, with possible implications during popular park hours. All parks contained benches that were in an excellent condition, and a high percentage of the park segments had garbage cans and signage. Another positive feature was that none of the path segments were under construction or had temporary barriers placed. 

Some features of parks that promote physical activity among adults were better or more readily available in some parks as compared to others. For example, although four of the five parks contained exercise or play equipment, only two parks contained exercise facilities for adults. The presence of slope in the path and the presence of litter on the trail was observed in some parks but not in others. Two of the five parks had path segments with services available (e.g., food or drinks available for purchase). None of the parks had drinking fountains, nearby transit stops, or emergency call boxes. 

Results regarding wheelchair accessibility were not included as they are presented elsewhere [59]. No dogs were present in any of the parks. Although not included as a question in the original audit tool, the number of cats was recorded as they are commonly found in public parks in Saudi Arabia. 

**Table 1:** Park features across five parks in Riyadh.

Measure	Park 1 Alwaha [60] N = 6	Park 2 Flower [61] N = 1	Park 3 Prince Abdulaziz [62] N = 4	Park 4 Alnada [63] N = 5	Park 5 Olaya [64] N = 3	Frequency Across All Segments
						Yes (%)
Intersecting road (proportion of segments with attribute)						
Signal or sign	1.0				0	66.9
Curb cut	0.83				0	55.6
Crosswalk	1.0				0	66.7
Raised crosswalk	1.0				0	66.7
Pedestrian signal	0				0	0
Trail design (proportion of segments with attribute)						
Suff. vert. clearance	1.0	1.0	1.0	1.0	1.0	100
Presence of shoulder	1.0	0	1.0	1.0	1.0	94.7
Adjacent road	1.0	1.0	1.0	1.0	0.33	89.5
Buffer from road	1.0	1.0	1.0	1.0	1.0	100
Access points	1.0	1.0	0.75	0.80	1.0	89.5
Gate or bollard	1.0	1.0	1.0	0.80	1.0	94.7
Viewpoint	0	1.0	1.0	0.80	1.0	63.2
Trail amenities (proportion of segments with attribute)						
Lights	1.0	1.0	1.0	1.0	1.0	100
Restrooms	0.17	1.0	1.0	0.40	1.0	57.9
Benches	0.83	1.0	1.0	0.80	1.0	89.5
Picnic tables	0	1.0	0.50	0	1.0	31.6
Drinking fountain	0	0	0	0	0	0
Garbage cans	0.50	1.0	1.0	1.0	1.0	84.2
Signs	1.0	1.0	1.0	0.80	1.0	94.7
Parking	0.50	1.0	1.0	1.0	1.0	84.2
Bicycle racks	0	0	0	0	0	0
Exercise/play equip	0.33	0	0.50	0.20	1.0	42.1
Exercise equipment	0.17	0	0	0	1.0	21.1
Services	0	1.0	1.0	0	0	26.3
Transit stops	0	0	0	0	0	0
Cultural institution	0.50	1.0	0.25	0.20	1.0	47.4
Com. destinations	0	1.0	0.50	0	0	15.8

Note: N = number of segments; suff. vert. clearance = sufficient vertical clearance; services = food service, gift shop, information, and bike rental; exercise/play equip = exercise or play equipment; and com. destination = commercial destinations. Values provided = proportion of segments with the feature listed present.

**Table 2:** Park features for ordered categorical measures across five parks in Riyadh.

Measure	Park 1 Alwaha [60] N = 6	Park 2 Flower [61] N = 1	Park 3 Prince Abdulaziz [62] N = 4	Park 4 Alnada [63] N = 5	Park 5 Olaya [64] N = 3	All Park Segments N = 19
Trail: Design M (SD)						
Path condition	3.3 (0.5)	5	5 (0)	2.6 (0.9)	4 (0)	3.7 (1.1)
Slope	1 (0)	2	1 (0)	1 (0)	1 (0)	1.1 (0.2)
Cross slope	1 (0)	2	1 (0)	1 (0)	1 (0)	1.1 (0.2)
Site distance	1 (0)	1	1 (0)	1 (0)	1 (0)	1.0 (0)
Lateral visibility	1.5 (0.6)	1	1 (0)	2 (0)	1 (0)	1.2 (0.4)
Width of buffer from road	2 (0)	2	2 (0)	2 (0)	2 (0)	2.0 (0)
Trail: Amenities M (SD)						
Bench condition/cleanliness	5 (0)	5	5 (0)	5 (0)	5 (0)	5 (0)
Picnic table condition/clean		4	4 (0)		3 (0)	3.5 (0.5)
Restroom cleanliness	1.0 (0)	2	1.5 (.6)	3 (0)	1 (0)	2.0 (0.8)
Garbage cans overflowing	1.7 (0.6)	1	1 (0)	1 (0)	1 (0)	1.1 (0.3)
Number of parking spots	1.5 (1.6)	3	4 (0)	1 (0)	4 (0)	3.8 (2.9)
Trail Maintenance M (SD)						
Glass	1.2 (0.4)	1	1 (0)	1 (0)	1 (0)	1.1 (0.2)
Litter	2.8 (1.0)	3	1 (0)	2 (0)	3 (0)	2.3 (0.9)
Graffiti	1 (0)	1	1 (0)	1 (0)	1 (0)	1.0 (0)
Vandalism	1.7 (0.5)	1	1 (0)	1 (0)	1 (0)	1.2 (0.4)
Odor	1 (0)	1	1 (0)	1 (0)	1 (0)	1.0 (0)
Noise	2.3 (0.8)	2	2 (0)	1.2 (0.4)	2 (0)	1.9 (0.7)
Animal droppings	1.7 (0.5)	1	1 (0)	1.2 (0.4)	1 (0)	1.3 (0.5)
Cats	0.7 (0.8)	7	1.5 (1.3)	1.0 (1.7)	7 (0)	2.3 (2.7)

N = number of segments; M = mean; SD = standard deviation. Path condition; bench condition/cleanliness; picnic table condition/cleanliness; restroom cleanliness: 1 = very poor; 2 = poor; 3 = fair; 4 = good; 5 = excellent. slope; cross slope: 1 = flat or gentle; 2 = moderate; 3 = steep. Width of buffer: 1 = < 1 meter; 2 = 1–3 m; 3 = > 3 m. Number of parking spaces: 1 = 10 or less; 2 = 11–25; 3 = 26–50; 4 = more than 50. Glass; litter; graffiti; vandalism; odour; noise; animal droppings: 1 = none; 2 = a little; 3 = some; 4 = a lot. Cats = number.

**Table 3:** Park’s location and size.

Park Name	District	Size (Sqm)
Alwaha Park [60]	King Salman District	6750
Flowers Park [61]	AlMohammadia	8000
Prince Abdulaziz Park [62]	AlHamra	75,960
AlNada Park [63]	AlNada District	Not Available
AlOlaya Park [64]	AlMathar Alshamali	37,000

## 4. Discussion

The purpose of this study was to examine the feasibility and utility of the PEAT for use in a larger-scale study focused specifically on older adults and to conduct a preliminary assessment of paths in five public parks. Results reveal that the PEAT measure [51] can be easily used by occupational and physical therapists to conduct systematic audits of walking paths in public parks. In future, OT students can be trained to conduct the audits in pairs, using GPS and a tablet computer as data collection aids. Noteworthily, due to the time-consuming nature, significant manpower is needed to audit all segments of a park. To use the tool in a larger-scale study, multiple trained team members will be needed. The tablet computer was a helpful resource as it allowed the input of findings directly into an Excel spreadsheet organized by park location and content area (i.e., path design, path amenities, path maintenance/aesthetics, and intersecting roads). Direct input into the Excel spreadsheet may have also minimized errors by avoiding having to transcribe the results from a hard copy/paper version of the instrument. The hand-held GPS App, Strava, served as a valuable resource to keep track of path segments [65]. Further, the screenshots of the GPS maps enabled auditors to easily record findings related to path/trail lengths. 

Although previous studies on park preferences among older adults have not specifically targeted individuals with chronic diseases, they do shed light on the types of parks features that may be attractive to older adults to promote park use. Examples include nature, water features, seating, flat walking surfaces that are in good condition, car parking, information signs, peaceful well-maintained spaces, multigenerational features, shading, and locations away from traffic [44,45]. Noteworthily, the PEAT captures most of these preferences except for shaded walkways, shaded areas for resting, and elements of nature other than viewpoints. Considering the five parks examined in this study, seating, flat walking surfaces, buffer areas from traffic, parking, and informational signs were almost universal. However, viewpoints and play/exercise equipment were only present in four of the parks, and the maintenance of spaces was varied. Moreover, while some parks were noted to have multiple available benches, they were all placed near the start of each segment and therefore may not be suitable for older adults with chronic diseases to have frequent rest breaks during their walks.

Although the PEAT was designed to examine features of parks that promote physical activity in general [43,51], some park features included in the measure are more relevant than others regarding the physical activity needs of older adults with chronic diseases. For example, bathrooms, path slope, lighting, litter, drinking fountains, and path conditions are all included in the measure. Given that people with heart disease are often prescribed diuretics [66], the availability of bathrooms is important for this population group. Litter can potentially be a trip/slip hazard, and cats can also present a risk for falls, especially among older adults [67]. A slope in the walking path could create a challenge for someone with heart disease who has a significant limitation in endurance/activity tolerance. The suggested guidelines for the length of segments imply that the concentration of benches that may be needed by some older adults with decreased activity tolerance are not being captured. Therefore, going forward, shorter segments as a unit of analysis are suggested for projects focused on older adult park users. Services, such as food available for purchase, are included in the PEAT. However, questions related to the availability of healthy food and beverage choices are not included in the PEAT. Therefore, questions in the audit about food and beverage choices relevant to cardiac prudent or diabetic diets should be added. 

To date, the focus of outdoor environmental assessment has been on the accessibility of playgrounds for children, walkability of neighbourhoods, and wheelchair accessibility of national parks [25,50,68,69]. Therefore, the assessment of features of public parks related to accessibility and usability for adults with chronic diseases warrants further attention. This study has set the stage for future research in this area of inquiry. 

Consideration of the cultural relevance of assessments and measurement tools is called for in research and clinical practice [70]. Although most features of the audit tool were appropriate for a Saudi Arabian context, some were not. For example, emergency call boxes are not used in Saudi Arabia as citizens now have access to a mobile phone app to request emergency services, for example, via Tawakkalna App [71]. Also, dogs are not popular pets in the Middle East generally, and laws in Saudi Arabia prohibit them in public parks except in dog-friendly parks and some pet-friendly cafes. Adequate lighting, consistent across the parks, is an important feature given that evening hours are the peak hours for park use in hot weather climate areas. Noteworthily, the presence of shaded places for resting is not included in the PEAT measure, yet the presence of such features could boost physical activity in parks that are situated in hot climates. On the other hand, one of the features that makes the audit tool efficacious in Saudi Arabia is assessing the presence of religious buildings along the trail segment, as this may motivate the user to walk in the park between or after prayer time and provide an alternative option to using one of their bathrooms if the park bathrooms are not appropriate. 

Self-management programs include incorporating physical activity into one’s habits and routines [72]. However, depending on an older adult’s impairments, some environments may be less conducive to walking outdoors. Therefore, the PEAT can be used to assist people with physical activity goal setting regarding identifying an appropriate location for walking outdoors based on both their preferences and physical needs. For example, using our results (i.e., five public parks in Riyadh) as an example, for someone with heart disease who has limited activity tolerance and requires frequent bathroom breaks, Prince Abdulaziz Park would be the best location for outdoor walking, given the flat slope, good condition of its paths, and the availability of both benches and bathrooms. However, further study is warranted to assess the utility of audit findings for individual goal setting. 

Limitations of this feasibility study include the small sample of parks in Riyadh. However, noteworthily, there was no single database available to provide sufficient information about the parks. Consequently, the researchers had to rely on multiple government resources as well as site visits. Another limitation pertains to the assessment of trail maintenance. Audits were conducted at one point in time; therefore, it is possible that maintenance varies depending on the day of the week and time of day. 

Despite limitations, the current study fills a gap regarding an assessment of accessibility and physical activity-promoting features of outdoor spaces, specifically public parks in Riyadh. The preliminary audit of paths in the five parks revealed that, overall, they contained numerous features that promote physical activity participation. Exceptions include the lack of key features such as water fountains, clean restrooms, and services. Although the data collection took place in one city in the Middle East, the study findings may have relevance beyond the five parks assessed in this study. Specifically, our findings suggest the need to assess park paths and trails for a higher concentration of benches, the need for inclusion of a question related to shaded places for walking and resting (especially in hot weather cities), additional questions pertaining to the presence of nature, and an item about the availability of food choices for those on restricted diets in future projects that focus on the physical activity needs of older adults. Also, in future projects, context-specific cultural relevance would need to be established for the audit if it is to be used in other global sites. 

Direction for future study includes audits of additional parks in strategically selected districts to serve as a resource for chronic disease self-management programs. Qualitative research incorporating the experiences of adults with chronic diseases regarding public park use is also called for. The Ecology of Human Performance Model [23] serves as a valuable framework to guide future interdisciplinary projects related to evaluation and intervention pertaining to outdoor spaces. 

## 5. Conclusions

In conclusion, provided that sufficient resources are available, the PEAT is feasible and acceptable for use in a larger-scale occupational therapist-led study. However, additional audit items are warranted for better applicability to older adults with chronic diseases and those living in hot climates. Environmental modifications, the maintenance of existing parks, and accessible design characteristics for newly developed parks are needed to help promote physical activity participation among the growing older adult population. 

## Data Availability

Data are available upon request from the corresponding author.

## Supplementary Materials

The following supporting information can be downloaded at: https://www.mdpi.com/article/10.3390/healthcare12161572/s1, Figure S1: Prince Abdulaziz bin Mohammed bin Ayyaf Park (https://www.trfihi-parks.com/en, accessed on 25 July 2024); Figure S2: Al-Waha park (https://www.trfihi-parks.com/en, accessed on 25 July 2024); Figure S3: Al-Nada Park (https://www.trfihi-parks.com/en, accessed on 25 July 2024); Figure S4: Flowers Garden (https://www.urtrips.com/en/flowers-garden-riyadh/, accessed on 25 July 2024); Figure S5: Al-Olaya Park (https://www.safarway.com/en/property/olaya-park, accessed on 25 July 2024).
