# Supplementary material for: Assessment of Park Paths and Trails for Physical Activity Promotion among Older Adults in Saudi Arabia: Feasibility and Future Directions
Source: Healthcare (Basel). 2024 Aug 8;12(16):1572. doi: 10.3390/healthcare12161572 (PMC11353773; doi:10.3390/healthcare12161572)
Supplement: Supplementary file 1 [file healthcare-12-01572-s001.zip › healthcare-3150313-supplementary.pdf]

Supplementary file S1

Prince Abdulaziz bin Mohammed bin Ayyaf Park

Prince Abdulaziz bin Mohammed bin Ayyaf Park is located on the Eastern Ring Road between the 10th and 9th exit of the Al Hamra district. The area is 75,960 m2, which is an outlet for the people of Riyadh. There is a pedestrian walkway around the site that is 1800 meters long, and fountains are present at the main entrance on the Eastern Ring Road on the site of the show yard in the centre of the park. There is also a courtyard with an area of 2700 and can accommodate more than 1000 spectators. Further, there are 28 shaded sessions, 12 open sessions, 2 water courses, 39,000 m2 of green space, and multi-stage children's games.

Entertainment Elements: track; sports; sitting places; entertainment; restaurants; kids area; and festivals.

Main Elements: green areas and multiple seating areas.

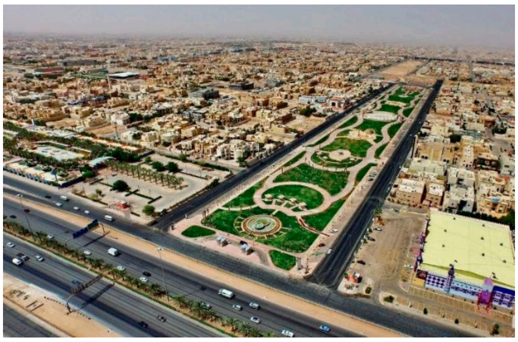

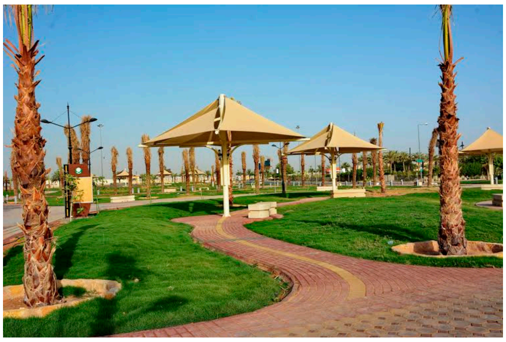

Figure S1. Prince Abdulaziz bin Mohammed bin Ayyaf Park (https://www.trfihi-parks.com/en).

AL Waha Park

Al Waha Park is a green oasis of tranquillity and relaxation. Modern facilities are well maintained in the Al Waha Quarter. With an area of 6,750 square meters, Al Waha Park has many attractions and facilities. Beautifully landscaped gardens, adorned with native plants, and attractively maintained footpaths are among the main features. The park is surrounded by a walkway that is approximately 2 kilometres long. At the main entrance, grand gates welcome visitors. In the centre of the park, there is a large courtyard, which is used for public gatherings and events, and covers 1,500 square meters. The park is filled with shaded areas that provide sun protection, along with expansive grassy lands and gardens. There are multi-stage children's games scattered throughout the park, offering enjoyable spaces for kids to engage in play. The park's expanse provides ample space for walking and jogging, allowing visitors to move freely without any hindrance from crowds. Al Waha Park also organizes events and festivals all year round to add a cultural dimension to its natural charm.

Entertainment Elements: track; sports; sitting places; entertainment; and restaurants.

Main Elements: cleanliness; green areas; and open paths for walking.

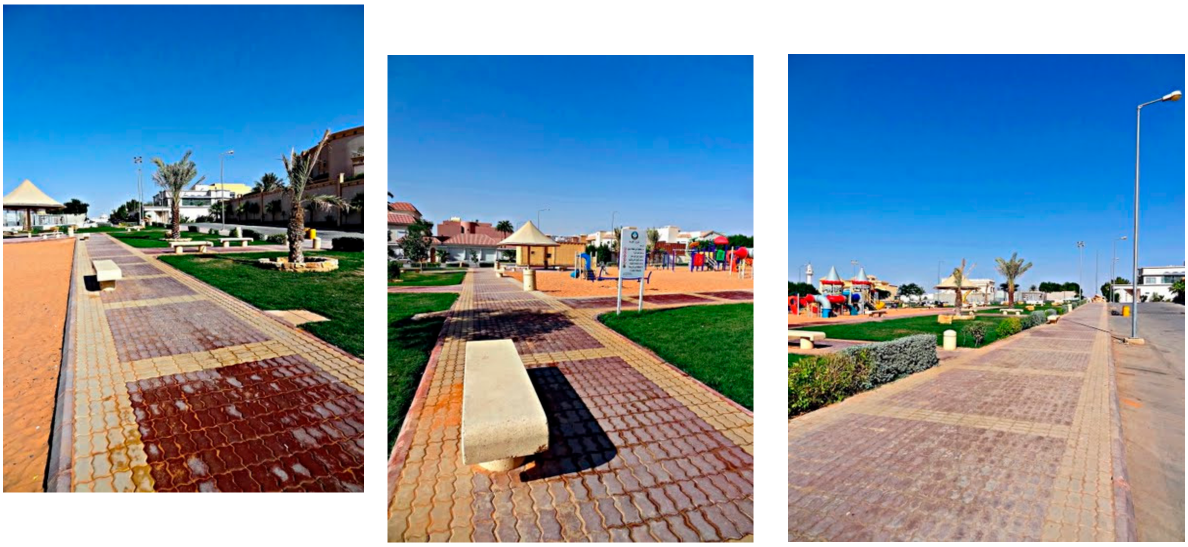

Figure S2. Al-Waha park (https://www.trfihi-parks.com/en).

Al Nada Park

Al Nada District Park in Riyadh is located centrally in the city, primarily constituting walking and jogging tracks encircling the site spanning an estimated 10,000 square meters within the main park land, and the landscaped pedestrian walkway around the site (1.5 km) offers a pleasant meander through green surroundings that park visitors can enjoy with brisk walking or jogging.

The welcoming park entrance serves as a gateway for all visitors. It is nicely landscaped and visually appealing, with signage welcoming guests. Groups using the centre can use the large courtyard at the heart of the park as a communal meeting space. The courtyard measures roughly 500 square meters and can host activities or performances in addition to offering a place for relaxation. The park boasts a wealth of green spaces, including cultivated gardens and abundant landscaping. Strategically placed multi-stage children's games offer a versatile experience for families with children of different ages.

Entertainment Elements: track; sports; sitting places; and kids area.

Main Elements: green areas; open paths for walking; and multiple seating areas.

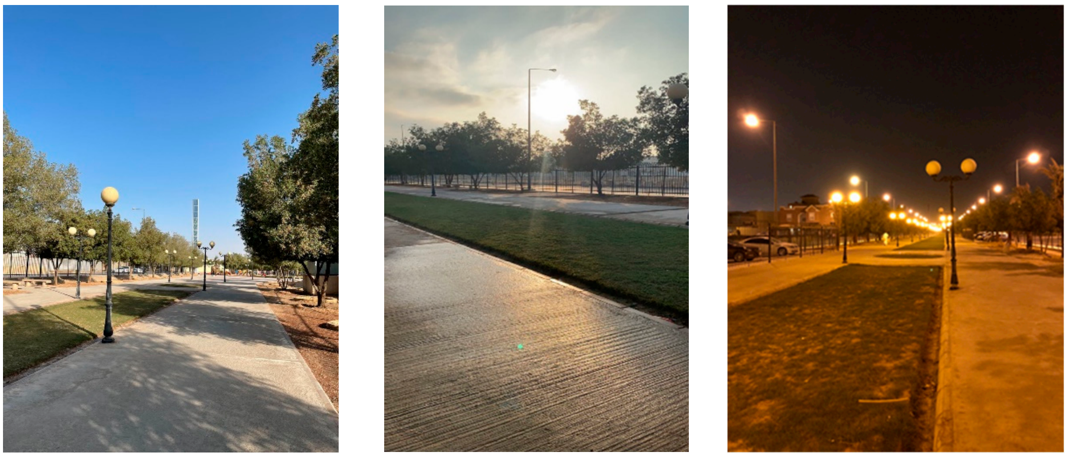

Figure S3. Al-Nada Park (https://www.trfihi-parks.com/en).

Flowers Garden

Flowers Garden in Riyadh includes more than 2 km of pedestrian walkways around the park, providing a visually appealing and beautiful path for visitors to enjoy. An impressive graphic gateway with floral elements welcomes visitors to one of the main entrances, setting a precedent for what is inside. A beautifully designed fountain is one of the focal points within this event space, providing both visual architecture and peaceful watery sounds. The open lawn's central park has a wide, fluid-reduced grass green area for leisure and sports use, with a 4,000-square-meter yard designed to set off different activity styles. This courtyard features sections that provide ample shade, as well as open areas that adapt to the prevailing weather conditions. The park is full of green areas, and there are various levels in the children's playground that cater to different ages for kids' safety, making it more fun.

Entertainment Elements: track and sitting places.

Main Elements: green areas; open paths for walking; and multiple seating areas.

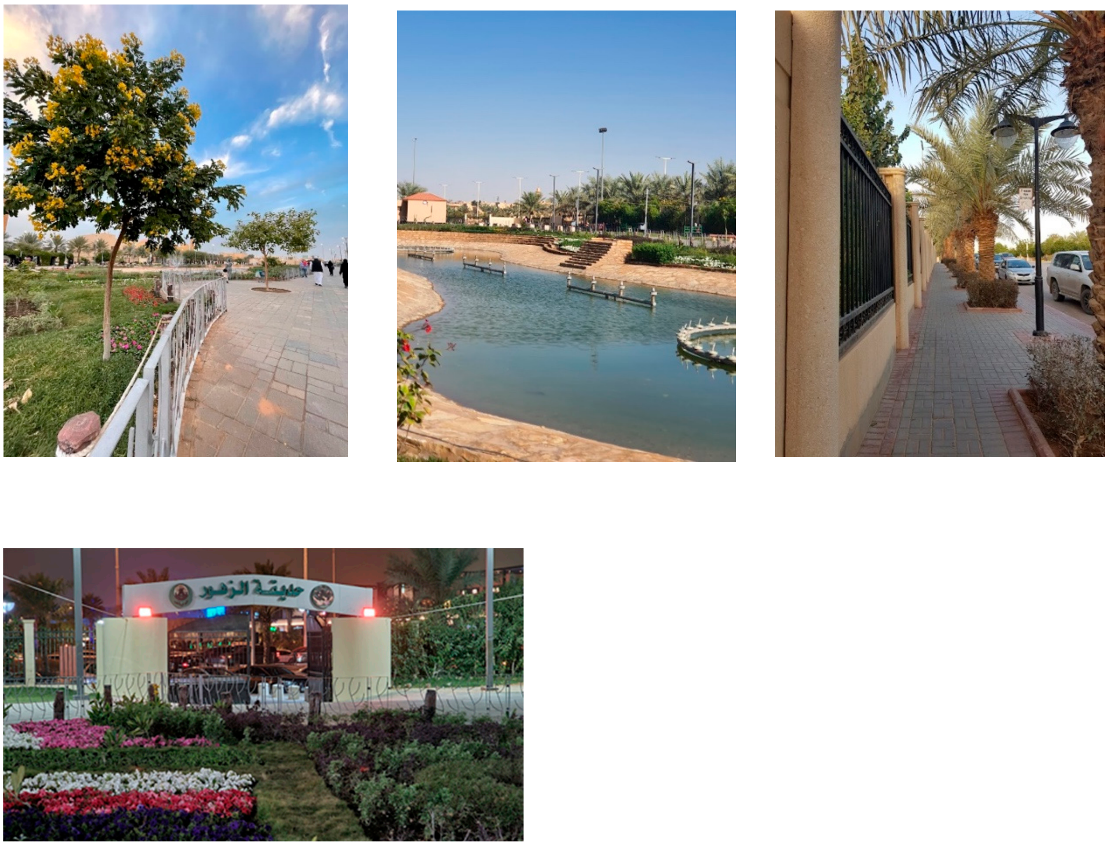

Figure S4. flowers Garden (https://www.urtrips.com/en/flowers-garden-riyadh/).

Al Olaya Park

Al Olaya Park in Riyadh spans an approximately 3,900 square meter area, which places significant emphasis on a pedestrian walkway penned along attractive traces that pass through the park from many sides up to the covered facilities.

The central fountain creates a focal point and provides soothing sounds of flowing water. Specifically, in the park's core, there are seating areas and open lawns. The park's front features a spacious plaza that offers adaptable space for various activities and events. Landscapers have integrated shaded areas, such as pergolas, trees, and

canopies, to complement the open spaces and cater to a variety of preferences. These areas provide refuge from direct sunlight between large lawns, making them ideal for picnics and outdoor sports. The park has plenty of landscaped green spaces characterized by manicured lawns, floral beds, and shrubbery to provide an attractive environment while promoting environmental friendliness. The park also boasts a spacious children's play area, equipped with a variety of interconnected activities and equipment suitable for various age groups and skill levels, ensuring the safety and enjoyment of all children.

Entertainment Elements: track and sitting places.

Main Elements: green areas; open paths for walking; and multiple seating areas.

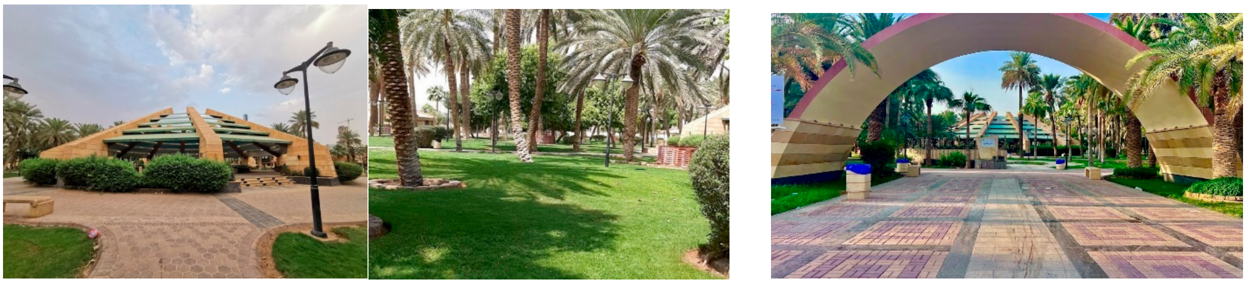

Figure S5. Al-Olaya Park (https://www.safarway.com/en/property/olaya-park).
